# Clinical and epidemiological aspects of complicated malaria in Colombia, 2007–2013

**DOI:** 10.1186/s12936-016-1323-5

**Published:** 2016-05-10

**Authors:** Pablo E. Chaparro-Narváez, Mary Lopez-Perez, Lina Marcela Rengifo, Julio Padilla, Sócrates Herrera, Myriam Arévalo-Herrera

**Affiliations:** National Institute of Health of Colombia, Bogotá, Colombia; Caucaseco Scientific Research Center, Cali, Colombia; Ministry of Health and Social Protection of Colombia, Bogotá, Colombia; Faculty of Health, Universidad del Valle, Cali, Colombia

**Keywords:** Complicated malaria, Malaria surveillance, Colombia, *Plasmodium falciparum*, *Plasmodium vivax*

## Abstract

**Background:**

During the last decade, Colombia presented a significant decrease in malaria clinical cases and associated mortality. However, there is a lack of reliable information about the prevalence and characteristics of complicated malaria cases as well as its association with different *Plasmodium* species. A description of the epidemiological and clinical aspects of complicated malaria in Colombia is presented here.

**Methods:**

A descriptive study was conducted using data collected between 2007 and 2013 by the Public Health Surveillance System (SIVIGILA). Demographic and clinical features were described. Frequency of complicated malaria cases, annual parasite index (API) and annual percent change (APC) for trend modelling by gender and age were also calculated.

**Results:**

A total of 547,542 malaria cases were recorded by SIVIGILA during the study period, of which 2553 (0.47 %) corresponded to complicated cases with similar distribution by *Plasmodium vivax* and *Plasmodium falciparum* species. Mixed infections were found in 153 cases (6.0 %). Trend modelling of the API for complicated malaria for all parasite species showed a non-significant increase throughout the years (APC 14.4 %; 95 % CI −4.3 to 36.6 %). Complicated malaria individuals were mostly males (62.2 %) and young adults (median age of 23 years). Notably, 72.4 % of the patients attended for malaria diagnosis >72 h after symptoms onset and 17 % reported malaria episodes in the last 30 days. All patients received anti-malarial treatment, but only 40 % received the first-line as recommended by the Colombian guidelines. Overall, hepatic and renal complications were the most common severe manifestations (63.6 %). Whereas hepatic and pulmonary complications were more common in *P. vivax* infections, renal and cerebral complications were significantly more frequent in patients with *P. falciparum*. In contrast with mono-infected patients, severe anaemia and shock were more frequent in patients with mixed infection.

**Conclusion:**

In contrast with the malaria-decreasing trend over the last years, the complicated malaria trend showed a non-significant annual increase. Therefore, in addition to existing national policies on early diagnosis and prompt anti-malarial treatment, more efforts have to be committed addressing the delayed diagnosis and inadequate treatment found in this study. Improving malaria notification forms, medical assistance skills, and capacity should be prioritized.

**Electronic supplementary material:**

The online version of this article (doi:10.1186/s12936-016-1323-5) contains supplementary material, which is available to authorized users.

## Background

Malaria continues to be an important public health problem in the developing world with 214 million clinical cases and 438,000 deaths estimated worldwide in 2015. *Plasmodium falciparum* is the predominant species worldwide responsible for ~94 % of malaria cases [1]. Conversely, in the Americas region, where malaria transmission is typically defined as hypoendemic and unstable (annual parasite index, API < 0.1 per 1000 per year) about 71 % of malaria cases are due to *Plasmodium vivax* [1]. In 2014, Colombia was the third most endemic country in Latin America (LA) and reported 17 % of the total 390,000 malaria cases [1]. Nonetheless, the country has displayed a malaria decreasing trend in prevalence and mortality the last decade; from 121,629 cases and 87 deaths in 2005 to 40,763 cases and 17 deaths in 2014 [1]. However, there is no reliable information about trends regarding complicated malaria cases.

Malaria exhibits a broad spectrum of clinical manifestations, including asymptomatic infection, uncomplicated and complicated malaria. This spectrum depends on multiple parasite, host and environmental factors [2–4]. Whereas in areas of high malaria transmission, individuals continuously exposed to *Plasmodium* develop partial protection for severe symptoms at an early age [4]; under conditions of low and unstable malaria transmission, individuals of all age groups may present with acute or severe disease as a consequence of low levels of naturally acquired immunity, although also subclinical infections are detected [4, 5].

In Colombia, a high frequency of uncomplicated *P. vivax* and *P. falciparum* malaria was reported recently [6], which appears to correlate with the relatively low malaria transmission and early diagnosis [7]. However, only a few studies have specifically focused on the description of complicated malaria cases in Colombia [8–14]. Before 2010, the criteria used for defining severe malaria in Colombia were the same established by the WHO for severe falciparum malaria [15], which were defined based on high transmission areas. In 2010, the Colombian Ministry of Health (MoH) adapted these criteria to the Colombian population, establishing more conservative parameters of complications to improve the detection and ensure a more effective treatment of these cases (Table 1).

**Table 1:** Malaria-related complications

Criteria	Defined before 2010 [22]	Defined after 2010 [21]^a^
Cerebral malaria	Impaired consciousness or coma (Blantyre score < 3 or Glasgow score < 9); unconsciousness with the possibility of waking up	Unchanged
Renal dysfunction	Serum creatinine > 3.0 mg/dL and/or urine vol < 400 mL in 24 h (adults) or <12 mL/kg of body weight in 24 h (children)	Serum creatinine > 1.5 mg/dL
Hepatic dysfunction	Serum bilirubin > 3 mg/dL and altered liver function tests	Serum bilirubin > 1.5 mg/dL or aminotransferases > 40 U/L
Respiratory distress	Increased respiratory rate at admission, presence of abnormal lung sounds or pulmonary oedema (X-rays)	Unchanged
Circulatory collapse or shock	SBP < 70 mm Hg in adults or <50 mm Hg in children (3–5 years)	SBP < 80 mm Hg in adults
Hyperemesis	>5 episodes in 24 h	Not applicable^b^
Hyperpyrexia	Axillary temperature >39.5 °C	Not applicable^b^
Hypoglycaemia	Blood glucose level < 40 mg/dL.	Blood glucose level < 60 mg/dL
Severe anaemia	Haemoglobin < 5 g/dL or haematocrit < 15 %	Haemoglobin < 7 g/dL
DIC	Abnormal bleeding in the presence of laboratory evidence of DIC	Unchanged
Acidaemia/acidosis and hyperlactemia	Acidaemia/acidosis (clinical signs)	Plasmatic bicarbonate < 15 mmol/L or base excess > −10; acidaemia pH <7.35; lactate acid > 5 mmol/L
Haemoglobinuria	Macroscopic haemoglobinuria	Macroscopic haemoglobinuria and positive urine dipstick
Hyperparasitaemia	>100,000 asexual parasites/μL of *P. falciparum* or in mixed infection with *P. vivax* and schizontaemia	>50,000 asexual parasites/μL

*DIC* disseminated intravascular coagulation, *SBP* systolic blood pressure

^a^Includes only the changes based on previous evidence

^b^Currently classified as a warning sign for complicated malaria development

In 2010, during a malaria outbreak in Colombia, 623 (0.5 %) complicated malaria cases were diagnosed among the 117,108 total cases reported to the Public Health Surveillance System (SIVIGILA) [16]. In this outbreak, the proportion of complicated malaria cases was similar for *P. vivax* and *P. falciparum,* with most patients (70.1 %) in the 15–64 years age group. In this and other studies [6, 17] hepatic and renal dysfunction were the most frequently reported complications. However, some studies have reported either hyperparasitaemia [10], severe anaemia [9, 17], or severe thrombocytopaenia [8, 13] as main malaria complications in Colombia.

All complicated malaria studies conducted in Colombia have been restricted to two of the most endemic areas in the country (Northwestern and Pacific regions), with many of them presenting gaps regarding epidemiological and clinical characteristics of complicated malaria cases [6, 8–13, 17–20]. Indeed, associations between *Plasmodium* species and clinical profile have been hardly explored [6, 8, 11, 12]. The aim of this study was to describe the clinical and epidemiological characteristics of complicated malaria in Colombia using national epidemiological records obtained by SIVIGILA from the whole country during the 2007–2013 period.

## Methods

### Study design and surveillance system

A descriptive study was conducted using data from the Colombian Public Health Surveillance System (SIVIGILA) for the 2007–2013 period. Briefly, all malaria cases including complicated and deaths cases are reported to the SIVIGILA by the primary data generating units (PDGU) and the information units (IU). The PDGU are the health institutions or points-of-care (POC) from both the private and the public system, where microscopists and physicians report to SIVIGILA. The IU are mobile or permanent malaria-diagnosis posts, where a trained and certified “primary agent” usually from the community is responsible for malaria diagnosis, notification and treatment of uncomplicated malaria cases. Malaria infection is confirmed by microscopic examination of Giemsa-stained thick blood smear (TBS) or by a positive rapid diagnostic test [21]. Uncomplicated malaria cases are reported on a weekly basis while complicated cases and deaths cases are reported on a daily basis [22].

### Case definitions

Case definitions were used as established by the Colombian MoH guidelines [21, 22]. A malaria case was defined by clinical malaria manifestations, i.e., history of fever and a positive TBS or RDT, regardless of the parasite species. Confirmed complicated malaria was defined as a malaria case with one or more of the clinical or laboratory parameters as described in Table 1. Complications are reported as it is discriminated in the SIVIGILA malaria notification forms: hepatic, renal, pulmonary and cerebral complication.

### Database mining and quality assurance

Databases were refined in agreement with SIVIGILA recommendations. Briefly, these recommendations included verification of data integrity, which means calculating the proportion of “empty data” and “no information data” for each study variable in the notification form. Only variables with integrity higher than 85 % were included in the study analyses. Integrity for demographic and epidemiological variables was higher than 99 %, whereas in some clinical and laboratory variables such as hyperemesis, shock, haemoglobin <5 g/dL and platelet counts <100,000 platelets/μL integrity was 88 %.

### Statistical analysis

Information was processed using Microsoft Excel spreadsheets and analysed using GraphPad Prism version 6.0 (GraphPad Software, San Diego, California, USA). The descriptive analysis included demographic and clinical variables. Univariate analysis was performed for all variables. Frequencies, measures of central tendency and dispersion were calculated. Chi square test was used to compare proportion differences. Odds ratios (OR) and 95 % confidence interval (95 % CI) were also calculated. A p value <0.05 was considered statistically significant.

The API for complicated malaria was calculated by relating the total number of complicated malaria cases with the population at risk multiplied by 100,000. Specific rates by 5-year age groups and gender were calculated and expressed as the number of cases per 100,000 individuals per year. The Joinpoint Regression^®^ software 4.0.4 (Surveillance Research Program, National Cancer Institute, Bethesda, USA) was used to calculated complicated malaria trends [23] adjusted by gender and API by 5-year age groups from 2005 national census [24]. Briefly, this method allows to identify the time point (year in this case) where a significant change happens and to estimate the magnitude of this change through the annual percent change (APC). A model using a maximum of three joinpoints was established to fits the simplest joinpoint model that the data allow. Heteroscedasticity was assumed and Monte Carlo Permutation method and Bonferroni correction were used as tests of significance. Trends were interpreted as (i) rising when 95 % IC of APC was >0 and statistically significant; (ii) decreasing when 95 % CI of APC was <0 and statistically significant; (iii) stable when 95 % CI of APC was between −0.5 and 0.5 and not statistically significant; (iv) non-significant when 95 % CI of APC was <−0.5 or >0.5 and not statistically significant. A p value <0.05 was considered statistically significant.

## Results

**Fig. 1:**
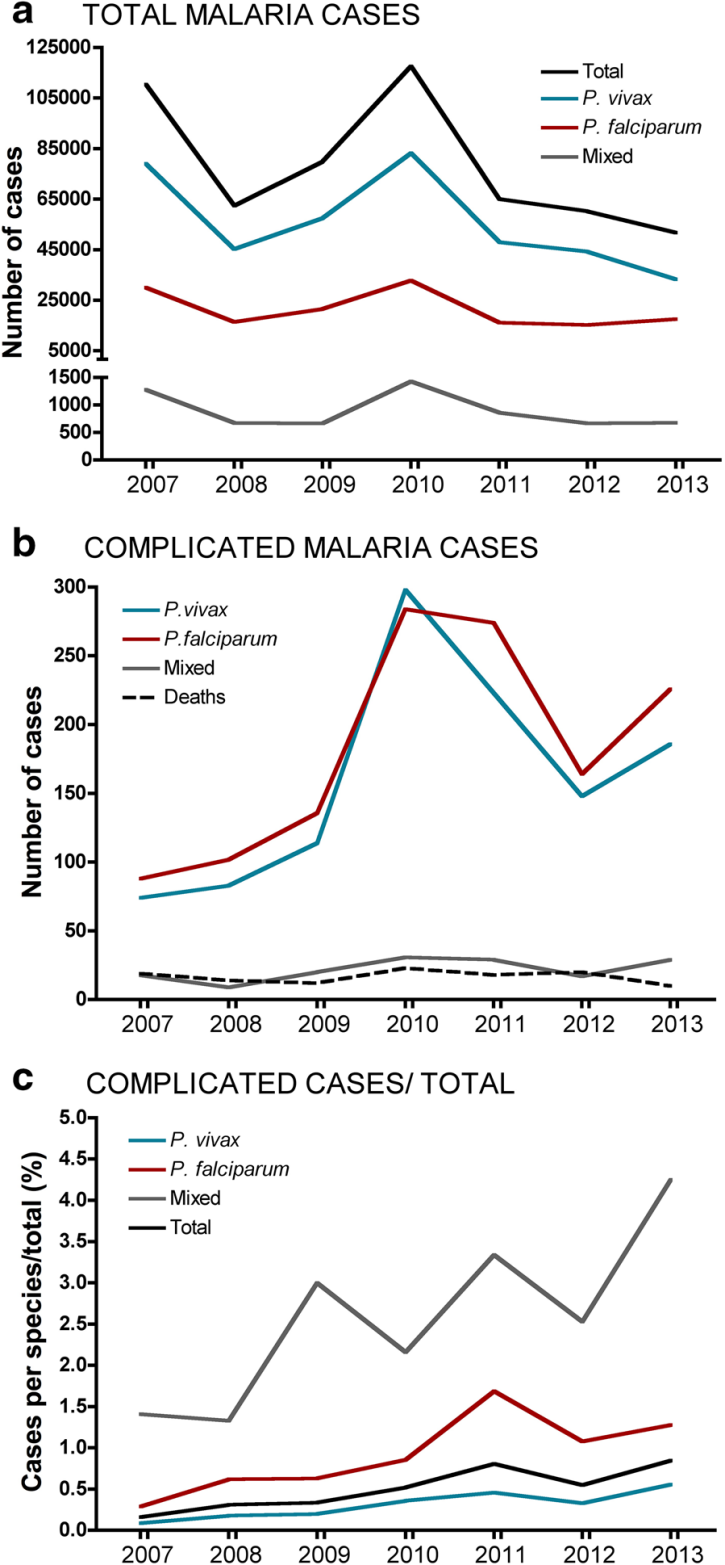
Malaria cases in Colombia. The number of total malaria cases (**a**) and complicated malaria cases (**b**) reported monthly between 2007 and 2013 are shown. *Black dashed line* (**b**), corresponds to the number of malaria-related deaths per year. **c** Percentage of complicated malaria cases over total cases according to the parasite species

Between 2007 and 2013, a total of 547,542 malaria cases were diagnosed and reported to SIVIGILA, with a notably decreasing trend over those years (Fig. 1a). While most of the cases corresponded to mono-infection caused by *P. vivax* (71.4 %) or *P. falciparum* (27.4 %), mixed malaria infection by both species were less frequent (1.2 %). During the same period, 2553 (0.47 %) complicated malaria cases were reported. A total of 1274 (49.9 %) of them were caused by *P. falciparum*, 1126 (44.1 %) by *P. vivax* and 153 cases (6.0 %) corresponded to mixed malaria infections (*P. falciparum* plus *P. vivax*; Fig. 1b). However, the average proportion of complicated over total cases per parasite species was higher for mixed infection than for *P. falciparum* (2.6 vs 0.9 %, respectively) and *P. vivax* (0.3 %; Fig. 1c). Of the total complicated cases, 1139 (44.6 %) were diagnosed between 2010 and 2011 (Fig. 1b). The highest numbers of cases were reported in the first semesters of 2010, 2011 and 2013 respectively, which corresponds with the average monthly highest incidence of malaria in Colombia, although transmission is reported throughout the year.

**Fig. 2:**
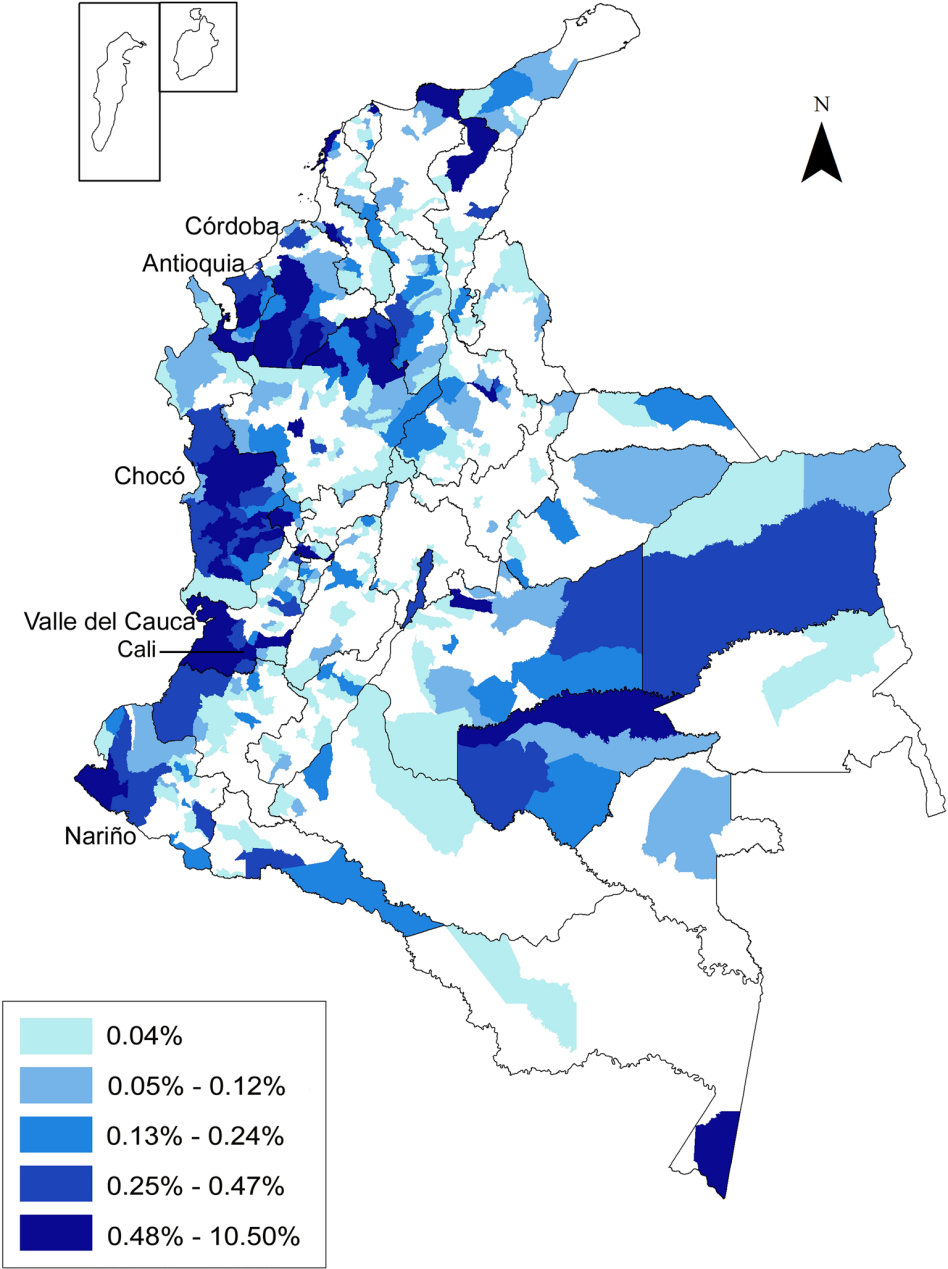
Distribution of complicated malaria cases in Colombia. Percentage ranges of complicated malaria cases between 2007 and 2013 are shown for different regions in Colombia. The five departments reporting the highest number of cases are shown

Most complicated malaria cases were recorded in the departments of Antioquia, Córdoba, Chocó, Nariño and Valle del Cauca (Fig. 2), all of them with endemic malaria transmission in several municipalities. Notably, four malaria-endemic municipalities in the Pacific Coast: Quibdó (Chocó), Buenaventura (Valle del Cauca), Istmina (Chocó), and Tumaco (Nariño) reported 21 % of the total complicated cases in the whole country. Moreover, cities without malaria transmission also reported complicated cases because patients from endemic municipalities are regularly referred to tertiary hospitals from other departments. Of those, Cali (Valle del Cauca) reported 7.1 % of the complicated cases, mainly referred from Buenaventura and Quibdó.

### Population characteristics

**Fig. 3:**
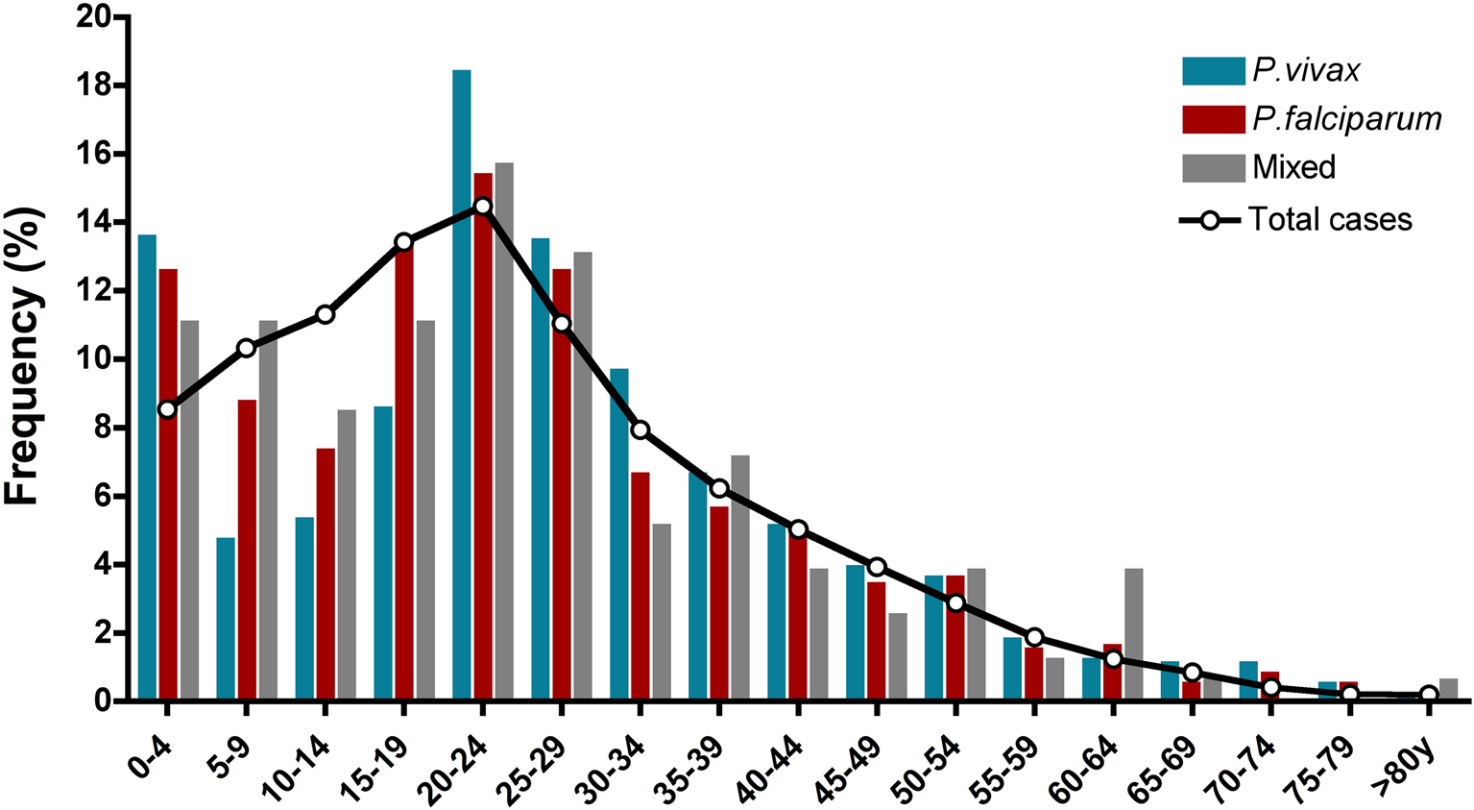
Parasite species distribution according to age. Percentage of complicated malaria cases with *P. falciparum*, *P. vivax* or mixed infections for 5-year age groups are shown. *Black line* corresponds to the percentage of total malaria cases (uncomplicated plus complicated) per group

Individuals with complicated malaria were mostly males (62.2 %; Table 2), with a median age of 23 years (range 0–94 years). The highest number of cases was reported in young adults between 20 and 29 years of age (n = 759, 29.7 %) and children younger than 5 years of age (n = 330, 12.9 %; Fig. 3). In children ≤15 years of age, mixed malaria was more frequent than single infections (p = 0.012; Table 2). Overall, patients presented late at health facilities for malaria diagnosis. Notably, 72.4 % of the patients attended for malaria diagnosis >72 h after symptoms onset (Table 2), a situation that was more frequent in the Pacific region (Additional file 1) and in patients with mixed malaria infection (p = 0.04).

**Table 2:** Characteristics of complicated malaria cases per parasite species

Variables	*P. vivax* (n = 1126)		*P. falciparum* (n = 1274)		Mixed infection (n = 153)		Total (n = 2553)		p value^a^
	n	%	n	%	n	%	n	%	
Gender									
Male	*734*	*65.2*	768	60.3	85	55.6	1587	62.2	*0.010*
Female	392	34.8	506	39.7	68	44.4	966	37.8	
Age									
≤15 years old	268	23.8	366	28.8	*47*	*30.7*	681	26.7	*0.012*
>15 years old	858	76.2	907	71.2	106	69.3	1871	73.3	
Time between symptoms onset and diagnosis									*0.044*
<24 h	149	13.2	129	10.1	13	8.5	291	11.4	
24–48 h	85	7.5	69	5.4	7	4.6	161	6.3	
48–72 h	110	9.8	129	10.1	13	8.5	252	9.9	
>72 h	781	69.4	947	74.3	*120*	*78.4*	1848	72.4	
No data	1	0.1	0	NA	0	NA	1	0.04	
Travel to malaria-endemic area in the last 15 days (yes)	*625*	*55.5*	536	42.1	52	34.0	1213	47.5	*<0.001*
Malaria episodes (last 30 days; yes)	219	19.4	187	14.7	*35*	*22.9*	441	17.3	*0.001*
Adequate anti-malarial treatment^b^	438	38.9	512	40.2	75	49.0	1025	40.1	ns
Blood transfusion (last 30 days; yes)	33	2.9	54	4.2	9	5.9	96	3.8	ns

Most frequent and significant data are highlighted in italics

*NS* non significant

^a^p value using the Chi square test between parasite species

^b^During the current episode

Seventeen percent of the patients reported malaria episodes in the last 30 days, mainly those with mixed malaria infection. Only 4 % of cases referred blood transfusion history in the last month. About half of cases (47.5 %) had a history of travelling to a malaria-endemic region during the 15 days prior to diagnosis, 37 % of them to a distant region from their residency, mainly to the Pacific and Urabá regions; a situation that was more common in *P. vivax*-infected patients (p < 0.001). Although all patients received anti-malarial treatment according to the Colombian guidelines [21], only 40 % of patients received first-line treatment for complicated malaria whereas the remaining 60 % received either second or third line of treatment (Table 2). Unfortunately, complaints such as poor compliance of either dose or duration of treatment cannot be ruled out, as those are not registered in the malaria notification forms.

### Trend modelling of the API

**Fig. 4:**
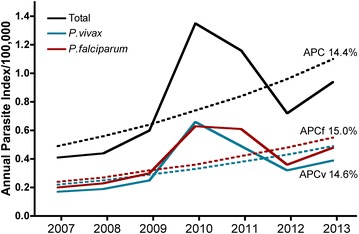
Trend of the API for complicated malaria. The observed (*solid lines*) and modelled (*dotted lines*) annual parasite incidence (API) for 100,000 habitants are shown. *APC* annual percent change

The API for complicated malaria was 0.8 for 100,000 (1.01 for male and 0.60 for female). Although some differences in the API were observed between *P. falciparum* and *P. vivax* infected patients (0.40 and 0.35 respectively) the APC for complicated malaria for both parasite species showed a non-significant annual increase in the whole population between 2007 and 2013 (14.4 %; 95 % CI −4.3 to 36.6). Similar trends were observed for *P. falciparum* (15.0 %; 95 % CI −1.7 to 34.7) and *P. vivax* (14.6 %; 95 % CI −7.8 to 42.5; Fig. 4). Age-stratified analysis (Additional file 2) showed a significant rising trend between 2007 and 2010 in the 0 to 4 years group (121.0 %; 95 % CI 8.4–350.5) and a stable trend between 2010 and 2013 (−13.0 %; 95 % CI −39.0 to 24.1). A rising trend between 2007 and 2013 was also observed for women, particularly in those infected by *P. falciparum* (Table 3).

**Table 3:** Annual percent change (APC) for complicated malaria, stratified by gender

Complicated cases	Male				Female				Comparison between genders	
	2007	2013	APC %	Trend	2007	2013	APC %	Trend	Test for parallelism^a^	Test for coincidence^b^
All parasite species	0.57	1.09	10.5	No change	0.25	0.80	19.9^c^	*Rising*	*<0.001*	*0.006*
*P. vivax*	0.27	0.51	10.4	No change	0.07	0.29	21.9	No change	*<0.001*	*<0.001*
*P. falciparum*	0.26	0.53	11.0	No change	0.14	0.44	20.5^c^	*Rising*	0.113	*<0.001*

Significant data are highlighted in italics

^a^A p value <0.05 is interpreted as the trends (slopes) between male and female are significantly different

^b^A p value <0.05 is interpreted as the rate differences (coincidence of intercepts) between male and female are significantly different

^c^The 95 % CI of annual percent change (APC) is significantly different of 0 (p < 0.05)

### Clinical findings

Some specific symptoms and signs were reported in complicated patients in addition to those frequently observed in acute malaria episodes (e.g., fever, headache, chills, and myalgias). Compared to patients with *P. vivax* mono-infections, those with *P. falciparum* had a higher risk of confusion (OR 1.76, 95 % CI 1.20–2.62), clinical jaundice (OR 1.60, 95 % CI 1.06–2.44), somnolence (OR 1.50, 95 % CI 1.17–1.93), and hepatomegaly (OR 1.34, 95 % CI 1.07–1.69). Interestingly, these manifestations were even more frequent in patients with mixed malaria infections (Table 4).

**Table 4:** Clinical findings in complicated malaria cases

Symptoms/signs	*P. vivax* (n = 1126)		*P. falciparum* (n = 1274)		Mixed infection (n = 153)		p value^a^
	n	%	n	%	n	%	
Hyperemesis	127	11.3	*193*	*15.1*	17	11.1	*0.010*
Hepatomegaly	160	14.2	232	18.2	*44*	*28.8*	*0.001*
Splenomegaly	128	11.4	150	11.8	*28*	*18.3*	*0.045*
Clinical jaundice	40	3.6	71	5.6	*11*	*7.2*	*0.024*
Somnolence	121	10.7	195	15.3	*28*	*18.3*	*0.001*
Confusion	44	3.9	85	6.7	*14*	*9.2*	*0.002*
Convulsions	39	3.5	65	5.1	*15*	*9.8*	*0.010*
Coma	14	1.2	6	0.5	0	0	0.180
Thrombocytopaenia^b^	494	43.9	545	42.8	*82*	*53.6*	*0.010*
Severe anaemia	84	6.9	117	8.3	*20*	*10.9*	*0.024*
Haemorrhage	54	4.8	42	3.3	5	3.3	0.390
DIC	19	1.7	17	1.3	5	3.3	0.190
Haematuria	43	3.8	69	5.4	*15*	*9.8*	*0.004*
Circulatory collapse or shock	31	2.6	12	0.9	*6*	*3.3*	*0.001*
Respiratory distress	56	5.0	62	4.9	10	6.5	0.870
Pulmonary oedema	22	2.0	19	1.5	5	3.3	0.540

Most frequent and significant data are highlighted in italics

*DIC* disseminated intravascular coagulation

^a^p value using the Chi square test between parasite species

^b^Non-severe thrombocytopenia defined as <100,000 platelets/µL

According to SIVIGILA classification, hepatic (33 %) and renal (31 %) complications were the most frequently presented regardless of parasite species. Renal (34.7 %) and cerebral complications (15.3 %) were significantly more common in patients with *P. falciparum*, while hepatic (37 %) and pulmonary complications (15.1 %) were more frequent in *P. vivax* cases (Table 5). Furthermore, a higher proportion of severe anaemia (10.9 %) and shock (3.3 %) was seen in patients with mixed malaria (Table 4).

**Table 5:** Frequency of organ-related complications

Complication	*P. vivax*		*P. falciparum*		Mixed infections		Total		p value^a^
	n	%	n	%	n	%	n	%	
Hepatic	*449*	*37.0*	418	29.8	55	30.1	922	33.0	*0.002*
Renal	313	25.8	*487*	*34.7*	56	30.6	856	30.6	*<0.001*
Pulmonary	*183*	*15.1*	138	9.8	17	9.3	338	12.1	*<0.001*
Cerebral	133	11.0	*214*	*15.3*	24	13.1	371	13.3	*0.002*

Total number of complications (according to SIVIGILA notification forms) taking into account that one patient can present more than one complication simultaneously

Most frequent and significant data are highlighted in italics

^a^p value using the Chi square test

### Malaria-related deaths

During 2007 and 2013, 116 (0.02 %) malaria-related deaths were reported (Fig. 1b). Those remained relatively low and decreased during the study period, with a mean of 17 ± 5 deaths per year and a mean of 2.2 ± 0.6 deaths per 10,000 total malaria cases. The malaria-related fatality rate (over all malaria cases) ranged between 0.02 and 0.03 %. Although a similar number of deaths was reported in mono-infections, the fatality rate per species was higher in those infected by *P. falciparum* than *P. vivax* (0.03 vs 0.01 %). Moreover, the fatality rate for mixed infection was particularly high (0.2 %).

## Discussion

Although Colombia has experienced a malaria-decreasing trend during the last decade [1], the observed trend for complicated malaria cases reflected a non-significant increase during 2007–2013 (APC 14.4 %; 95 % CI −4.3 to 36.6), with the highest number of cases reported after 2010 [16]. Notably, between 2007 and 2010 a significant rising trend for complicated malaria was found in children younger than 5 years of age and women mostly infected by *P. falciparum*. This could be explained by both the overall rise on the number of total malaria cases [16] and changes in complicated malaria criteria occurred in 2010. However, the proportion of total malaria cases in women compared to men remained similar during the study period. While changes in complicated malaria trend could be explained by a higher diagnosis rate due to more conservative definitions after 2010, improvement in the surveillance system and health care infrastructure should also be considered. Those data together suggest the need for new public health strategies focused on these vulnerable populations.

Complicated malaria represented 0.47 % of 547,542 malaria cases during 2007–2013, in agreement with a previous report of malaria outbreak using SIVIGILA information [16]. However, in a passive surveillance conducted between 2011 and 2013 was reported that 45 of 99 complicated malaria cases were not diagnosed and treated as such, but were classified during the laboratory data analyses [6], suggesting a possible underreporting of complicated malaria cases found here.

Although *P. falciparum* has been classically associated with a more severe clinical spectrum, there are multiple studies worldwide reporting an increasing number of severe manifestations in *P. vivax* infections [6, 9, 13, 17, 25–28], a relevant matter now on the malaria eradication agenda [29]. In this study, *P. vivax* was responsible for 44 % of complicated malaria cases, underscoring the importance of *P. vivax* malaria in a country where it causes about 70 % of malaria cases. Although the mechanisms involved in the clinical complications by *P. vivax* are not well understood [30, 31]; recent ex vivo studies have shown that *P. vivax*-infected red blood cells (*Pv*-iRBC) can adhere to endothelial cells [32–34], supporting the hypothesis that *P. vivax* undergoes sequestration in vivo.

Patients with mono-infections by *P. falciparum* had a higher risk of hepatic and neurological manifestations in comparison with *P. vivax* malaria patients. Non-severe thrombocytopaenia was found in almost half of complicated malaria cases, similar to a previous report in Colombia [6]. That is an important manifestation since altered platelet indices have been found as potential markers of severe malaria [18, 35]. Unfortunately, further information of severe thrombocytopaenia in patients studied here was not available.

Although severe anaemia was rather uncommon in mono-infections (~8 %), as also shown by other Colombian studies [6, 8, 10–13], renal and cerebral complications were significantly more common in patients with *P. falciparum* infections. In contrast, in patients with *P. vivax,* cerebral complications were less frequent (11 %), while hepatic and pulmonary complications were more common than in *P. falciparum* patients. The presence of other possible central nervous system pathogens has not been ruled out in this and other studies reporting malaria cerebral complications as reviewed in [28]. Indeed, it has been suggested that comma in *P. vivax* is 23 times less common than in *P. falciparum* infections [28]. The high number of cases with cerebral complications reported in this study contrasts significantly with previous reports from Colombia, with 1/92 complicated cases by *P.falciparum* [11], no cerebral malaria cases in 16 *P. vivax* complicated patients [13], and only one case of *P. vivax* cerebral malaria complicated with venous sinus thrombosis reported recently [36]. The contrasting high number of cerebral malaria cases reported to SIVIGILA, suggests an inappropriate classification probably due to errors during the filling or typing form process.

In contrast with mono-infected patients, complicated malaria was more frequent in patients with mixed infections, which presented commonly with hepatic and neurological manifestations, as well as severe anaemia and shock complications. Moreover, a high fatality rate was observed in those patients. Although some studies have suggested that *P. vivax* appears to attenuate the severity of the *P. falciparum* infection [37–39], others suggest that mixed infections could be associated with more severe disease, leading to a higher risk for severe anaemia, multiple organ dysfunction and mortality [12, 38, 40].

In agreement with other studies, a higher number of complicated malaria cases was found in young males [6, 16, 25, 28]; which could be explained by occupational factors such as mining, timber or farming that increase the risk of malaria infection. Notably, a diagnosis delay (>72 h after symptoms onset) was found in most patients, possibly due to either limited access to healthcare services (less developed on the Pacific Coast) or health staff failure to identify complicated malaria manifestations. Moreover, this delayed diagnosis increases the risk of anaemia [41]. Although a re-infection or relapse cannot be ruled out in patients reporting previous malaria episodes in the last month, which was significantly more common in cases with mixed infection, it suggests therapeutic failure or low adherence to anti-malarial treatment. Both delayed and the limited availability of the first line of anti-malarial drugs at health institutions could be responsible for higher parasite burden and secondary severe malaria.

These findings demonstrate the Colombian Health System faults in prompt recognition of complicated malaria patients as well as the difficulties around appropriate treatment provision; which may explain why the API for complicated malaria remained almost stable while API for total malaria cases decreased over the years. Although the databases were refined according to the national system, some data including demographic, epidemiological and clinical information were missed in less than 2 % of cases. Other constraints such as errors during the malaria notification form filling or typing process cannot be ruled out. Also, there may be misclassification of cases resulting from lack of adherence to case definitions, errors in species diagnosis -since molecular confirmation was not performed- and missing information about co-morbidities or co-infections. Although the Colombian guidelines use the same criteria to define complicated vivax and falciparum malaria [21], similar to the established by the WHO in 2014 [42]; the criterion of hyperparasitaemia must be re-evaluated in the Colombian guidelines since *P. vivax* invades reticulocytes and their parasite densities are usually lower than *P. falciparum*. Moreover, because the co-morbidities may modify the malaria episode course, this must be also included in the notification forms. Currently, the MoH is doing efforts in the reinforcement of health personnel knowledge and training about malaria disease and the correct notification process, especially in endemic areas with limited health infrastructure to strength the malaria information system in Colombia.

It is important to highlight that since the information reported here was obtained from the SIVIGILA, the data contained in the notification forms are the required for malaria surveillance and not for the clinical characterization. Thus, in this study malaria complications were presented as organ-related complications grouped in four categories (cerebral, pulmonary, hepatic, and renal complications) as appear in the SIVIGILA malaria case notification forms, instead of clinical and/or laboratory criteria of complications, as it is suggested by national and international guidelines. This categorization excludes several of the defined clinical and laboratory parameters but includes others in the notification forms under the clinical findings section. Therefore, parameters such as shock, haematological alterations, metabolic acidosis, haemoglobinuria, and hyperparasitaemia, which did not completely fit any of the four categories of complications, were excluded from the surveillance; suggesting considerable underreporting of total complicated malaria cases. Nevertheless, SIVIGILA malaria case notification forms were modified in 2016, reclassifying complications in six groups: cerebral, renal, hepatic, pulmonary, haematological, and other complication. Prospective studies that evaluate parasite (virulence, genetic profile, anti-malarial drug resistance) and host susceptibility factors (co-morbidities, co-infections, age of exposure, immunological and nutritional status) in conjunction with clinical and laboratory profile of complicated malaria patients should be conducted to improve existing knowledge of the clinical presentation spectrum and risks.

## Conclusion

During the last decade, Colombia presented a significant decrease in malaria clinical cases and associated mortality. However, it appears that the prevalence of complicated malaria remained stable. Despite existing national policies on early diagnosis and prompt anti-malarial treatment to prevent complicated malaria cases, more efforts have to be committed to decrease the total number of malaria cases, including the complicated ones. These efforts should include taking action to improve the malaria notification forms, medical assistance skills and capacity as well as to increase the knowledge of malaria-related risk factors, morbidity and mortality.

## Additional files

10.1186/s12936-016-1323-5 Time between onset of symptoms and malaria diagnosis. Percentage of complicated malaria cases from total cases reported is shown for each indicated Colombian region according to time between onset of symptoms and malaria diagnosis.
10.1186/s12936-016-1323-5 Annual percentage change (APC) for complicated malaria, stratified by age.

## Abbreviations

APC: annual percent change
API: annual parasitic index
iRBC: infected red blood cell
IU: information units
MoH: Ministry of Health
PDGU: primary data generating units
OR: odds ratio
POC: point-of-care
SIVIGILA: Public Health Surveillance System
TBS: thick blood smears
