# Supplementary figures and images for: Clinical and epidemiological aspects of complicated malaria in Colombia, 2007–2013
Source: Malar J. 2016 May 10;15:269. doi: 10.1186/s12936-016-1323-5 (PMC4863335; doi:10.1186/s12936-016-1323-5)

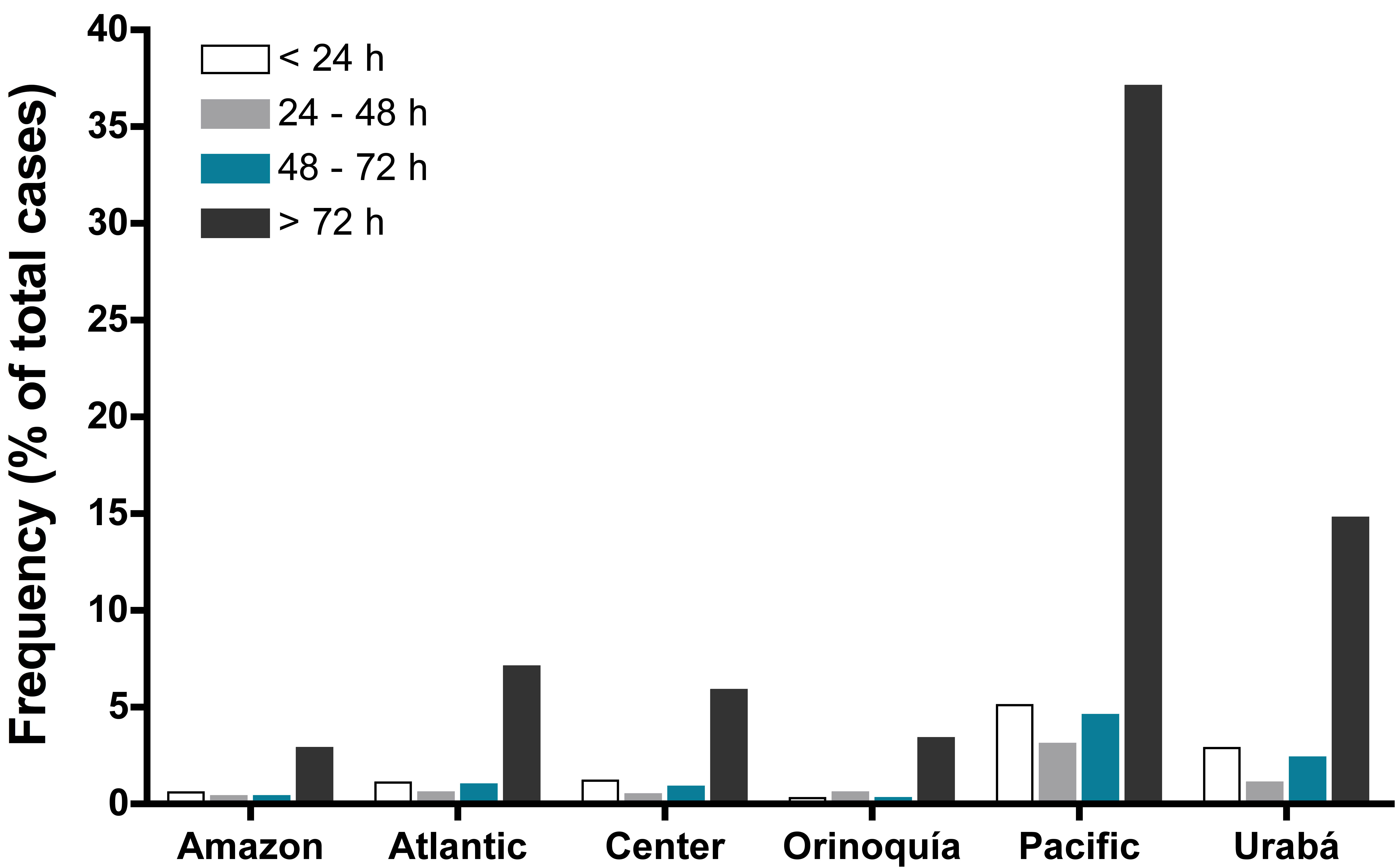

Supplement: Supplementary file 1 — 10.1186/s12936-016-1323-5 Time between onset of symptoms and malaria diagnosis. Percentage of complicated malaria cases from total cases reported is shown for each indicated Colombian region according to time between onset of symptoms and malaria diagnosis. [file 12936_2016_1323_MOESM1_ESM.tif]
