# Supplementary material for: Clinical and epidemiological aspects of complicated malaria in Colombia, 2007–2013
Source: Malar J. 2016 May 10;15:269. doi: 10.1186/s12936-016-1323-5 (PMC4863335; doi:10.1186/s12936-016-1323-5)
Supplement: Supplementary file 2 — 10.1186/s12936-016-1323-5 Annual percentage change (APC) for complicated malaria, stratified by age. [file 12936_2016_1323_MOESM2_ESM.doc]

**Clinical and epidemiological aspects of complicated malaria in Colombia, 2007-2013**

**Additional file 2.** Annual Percentage Change (APC) for complicated malaria, stratified by age.

| **Five-year age groups** | **Period** | **APC %** | **95%CI** | | **Trend** |
| --- | --- | --- | --- | --- | --- |
|  |  |  | **Lower** | **Upper** |  |
| 0-4 | 2007 - 2010 | **121.0*^a^*** | **8.4** | **350.5** | **Rising** |
|  | 2010 - 2013 | -13.0 | -39.0 | 24.1 | Stable |
| 5-9 | 2007 - 2010 | 91.1 | -44.8 | 562.4 | No change |
|  | 2010 - 2013 | -5.9 | -51.6 | 83.1 | No change |
| 10-14 | 2007 - 2013 | 12.3 | -17.6 | 53.1 | No change |
| 15-19 | 2007 - 2013 | 10.4 | -6.4 | 30.3 | No change |
| 20-24 | 2007 - 2013 | 2.2 | -19.8 | 30.3 | No change |
| 25-29 | 2007 - 2010 | 55.9 | -17.5 | 194.8 | No change |
|  | 2010 - 2013 | -16.4 | -47.4 | 32.8 | No change |
| 30-34 | 2007 - 2013 | 9.2 | -15.4 | 40.8 | No change |
| 35-39 | 2007 - 2013 | 13.3 | -4.4 | 34.2 | No change |
| 40-44 | 2007 - 2010 | 51.0 | -19.1 | 182 | No change |
|  | 2010 - 2013 | -17.9 | -49.5 | 33.2 | No change |
| 45-49 | 2007 - 2013 | 11.7 | -8.3 | 36.1 | No change |
| 50-54 | 2007 - 2013 | 12.8 | -27.0 | 74.4 | No change |
| 55-59 | 2007 - 2013 | 20.9 | -1.0 | 47.7 | No change |
| 60-64 | 2007 - 2013 | 16.0 | -10.5 | 50.3 | No change |
| ≥65 y | 2007 - 2013 | 11.5 | -0.1 | 24.4 | No change |

*^a^* Annual Percent Change (APC) significantly different from 0 (p<0.05); 95% CI, 95% confidence interval. Significant data are in bold.
